# The potential utility of CHATGPT4.0 as an AI assistant in the education and management of patients with Barrett’s esophagus

**DOI:** 10.1093/dote/doaf050

**Published:** 2025-07-07

**Authors:** Frances Dang, Josh Kwon, Andy Lin, Shoujit Banerjee, Trevor McCracken, Amirali Tavangar, Shravani R Reddy, Alyssa Y Choi, Jennifer Phan, Jeffrey D Mosko, Samir C Grover, Tyler M Berzin, Jason Samarasena

**Affiliations:** Division of Gastroenterology and Hepatology, University of California, Irvine, CA, USA; Division of Gastroenterology and Hepatology, University of California, Irvine, CA, USA; Department of Internal Medicine, University of California, Irvine, CA, USA; Department of Internal Medicine, University of California, Irvine, CA, USA; Department of Internal Medicine, University of California, Irvine, CA, USA; Division of Gastroenterology and Hepatology, University of California, Irvine, CA, USA; Division of Gastroenterology and Hepatology, University of California, Irvine, CA, USA; Division of Gastroenterology, VA Puget Sound Health Care System, Seattle, WA, USA; Advanced Endoscopy Program, Hoag Digestive Health Institute, Newport Beach, CA, USA; The Center for Therapeutic Endoscopy and Endoscopic Oncology, St. Michael’s Hospital, Toronto, ON, Canada; Division of Gastroenterology, Scarborough Health Network, Scarborough, ON, Canada; Center for Advanced Endoscopy, Beth Israel Deaconess Medical Center, Harvard Medical School, Boston, MA, USA; Division of Gastroenterology and Hepatology, University of California, Irvine, CA, USA

**Keywords:** accuracy, artificial intelligence, Barrett’s esophagus, Chatbot, gastroesophageal reflux disease, large language models

## Abstract

Chat Generative Pre-trained Transformer (ChatGPT) has emerged as a new technology for physicians and patients to obtain medical information. Our aim was to assess the ability of ChatGPT 4.0 to deliver high-quality information in response to commonly asked questions and management recommendations for Barrett’s esophagus (BE). Twenty-nine questions (14 clinical vignettes and 15 frequently asked questions (FAQ)) on BE were entered into ChatGPT 4.0. Using a 5-point Likert scale, three gastroenterologists with expertise in BE rated the 29 ChatGPT responses for accuracy, completeness, empathy, use of excessive medical jargon, and appropriateness to send to patients. Three separate gastroenterologists generated responses to the same 15 FAQs on BE. A group of blinded patients with BE evaluated both ChatGPT and gastroenterologist responses on quality, clarity, empathy and which of the two responses was preferred. Gastroenterologists rated ChatGPT responses as mostly accurate overall (4.01 out of 5) with 79.3% of responses completely accurate or mostly accurate with minor errors. When compared to gastroenterologist responses, the patient panel rated ChatGPT responses to be of significantly higher quality (4.42 vs. 3.07 out of 5) and empathy (4.33 vs. 2.55 out of 5) (*p* < 0.0001). In conclusion, ChatGPT 4.0 provides generally accurate and comprehensive information about BE. Patients expressed a clear preference for ChatGPT responses over those of gastroenterologists, finding responses from ChatGPT to be of higher quality and empathy. This study highlights the potential use of ChatGPT 4.0 as an adjunctive tool for physicians to provide real-time, high-quality information about BE to their patients.

## Graphical Abstract

**Graphical Abstract:**
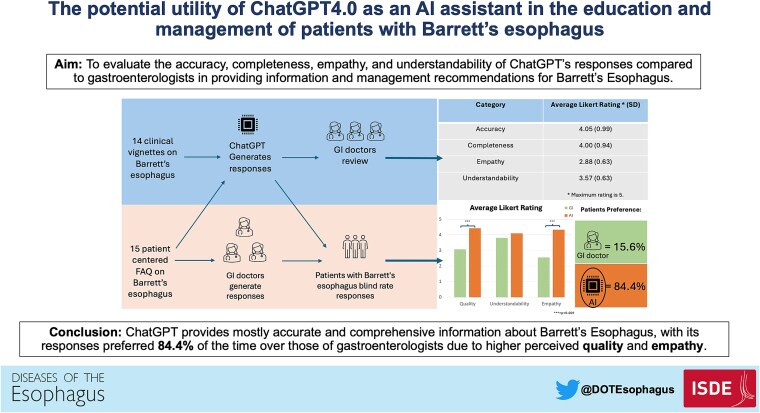

## INTRODUCTION

Barrett’s esophagus (BE) is a pathological disease process caused by chronic gastroesophageal reflux disease and is characterized by the replacement of squamous epithelium with metaplastic columnar epithelial tissue.^1–3^ BE is estimated to affect 1%–5% of people worldwide, with a greater predilection for older Caucasian males.^4^ Other risk factors include advanced age (>50 years old), smoking, obesity, and longstanding gastroesophageal reflux disease (GERD).^3^ It is estimated that 5%–12% of patients with longstanding GERD may develop BE, which is a strong predictor for the development of esophageal adenocarcinoma, as the natural course involves progression through multiple stages of dysplasia.^5^ As a premalignant and chronic medical condition, BE is a significant burden for patients and is associated with higher levels of depression and anxiety symptoms.^6^ Managing the complexities of patients with BE including different modalities for screening and prevention, treatment options, to surveillance can be time consuming, which, akin to many other GI diseases, has garnered interest in use of artificial intelligence (AI).

AI has made significant progress over the past few years, particularly in the realm of natural language processing.^7^ One manifestation of these advancements, Chat Generative Pretrained Transformer 4.0 (ChatGPT), has proven itself to be a useful clinical tool for communicating with patients by generating conversational text responses to a given prompt.^8^ The advent of this technology has made it a potential candidate to help facilitate physician–patient communication.^9–13^ Within the field of gastroenterology, ChatGPT satisfactorily answered straightforward questions about diagnosis and treatment options for GERD; however, with more difficult questions, ChatGPT failed to consistently provide medically accurate responses.^14^ Later studies corroborated these findings for eosinophilic esophagitis (EOE) and colorectal cancer screening.^15^^,^^16^ While ChatGPT was unable to pass the American College of Gastroenterology (ACG) examination, it successfully passed the United States Medical Licensing Examination (USMLE).^17^^,^^18^ This demonstrates that although ChatGPT can access and convey a significant level of medical knowledge, it has not yet been able to deliver consistent high-quality medical responses, particularly in the world of specialty medicine.

Therefore, it must be demonstrated that ChatGPT can reliably produce medically accurate responses that gain the approval of both physicians and patients. As excessive documentation and the medical inbox are key contributors to physician burnout, AI has the potential to reduce time spent responding to patient inquiries.^19^ Interestingly, it may even lead to greater patient satisfaction as one recent study showed that patients believe that AI-generated responses were more empathetic and of higher quality when compared to responses from human counterparts.^12^ The objective of our study is to evaluate the proficiency of ChatGPT in providing medical information and management recommendations regarding BE. Novel to this study, we will also assess the quality, clarity, and empathy of AI-generated answers to common patient-centered questions pertaining to BE. We hypothesize that ChatGPT will provide accurate and empathetic information about BE in both domains of commonly asked patient questions and clinically focused vignettes.

## METHODS

**Fig. 1:**
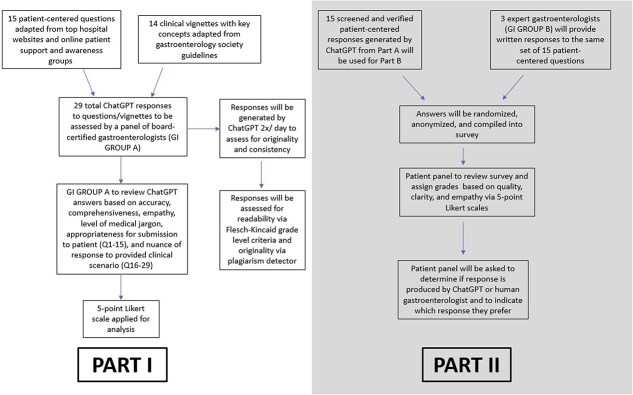
Study flowchart.

A total of 29 questions (15 patient-centered questions and 14 clinical vignettes) were input into ChatGPT 4.0 to generate responses in November 2023. The patient-centered questions (Q1–Q15) were derived from sections of Frequently Asked Questions of top US hospital centers and online Barrett’s patient support and awareness groups (Appendix IV). The clinical vignettes (Q16–Q29) were comprised of key clinical concepts regarding BE from updated gastrointestinal society guidelines (Appendix I). Of note, no additional prompting or guidance was provided when entering questions into ChatGPT. Each of the 29 questions was submitted twice on the same day into the ChatGPT interface to ensure consistency of responses. Figure 1 demonstrates the study protocol.

Part I of the study involves the evaluation of ChatGPT responses by a panel of gastroenterologists (JM, TB, SG) with expertise and training in BE. The expert gastroenterologists were asked to rate the responses for accuracy, completeness, empathy, and use of flowery or excessive medical jargon by means of 5-point Likert scales. For responses to the patient-centered questions (Q1–Q15), the panel was asked to rate their comfort level in sending these responses to a patient using a separate 5-point Likert scale. For the responses to the clinical vignettes (Q16–Q29), the panel was asked by means of another Likert scale to evaluate how nuanced and specific each response was to the provided clinical scenario (Appendix II). For all 29 questions, a free response comment section was provided where the panel was asked if they felt each response was ‘good’ or ‘bad’ overall and any additional comments.

Part II of the study compares the responses between ChatGPT and attending gastroenterologists from the patients’ perspective. Three board-certified attending gastroenterologists (AC, JP, SR) with expertise in BE were asked to generate responses to the patient-centered questions (Q1–Q15). The gastroenterologists were instructed to answer the questions as if they were responding to patient inquiries through a clinical messaging system (i.e. electronic health record, or EHR, inbox). The responses from both ChatGPT and gastroenterologists were then collected and randomly distributed to an anonymous, blinded panel of patients with a confirmed diagnosis of BE. For each question in the survey, there were two responses (one AI- and one gastroenterologist-generated) that were randomized in order to be presented. Using 5-point Likert scales, patients were asked to evaluate the overall quality and satisfaction with each response, the degree to which each response was easy to understand, and how empathetic or personable each response was. Patients were also asked if each answer was written by ChatGPT or a human gastroenterologist and to indicate which of the two answers they preferred. The Likert grading scales and questions used by the patient evaluators are shown in Appendix III.

### Data analysis

After the surveys were completed, data were compiled in Microsoft Excel and Stata. Descriptive statistics were performed. The average Likert scale rating and standard deviation for each survey category for both Part A and Part B were calculated. For Part B, the scores for each category (i.e. quality understandability, empathy) with respect to gastroenterologist and ChatGPT-generated responses were compared using a paired *T*-test to assess significance and confidence interval. Inter-rater reliability was assessed using Kendall’s coefficient of concordance. Originality of answers was evaluated using Copyleaks plagiarism detector. The Flesch–Kincaid grade level (formula: 0.39 × (words/sentence) + 11.8 × (syllables/words) − 15.59) was used to assess the readability of ChatGPT and gastroenterologist-generated responses. Statistical significance was defined as *p* < 0.05.

## RESULTS

**Fig. 2:**
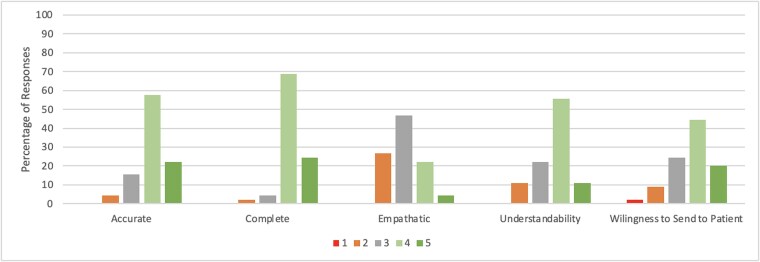
Expert rating of ChatGPT responses to patient-centered questions. † Likert scale ratings: *Accuracy:* 1—Completely inaccurate, 2- Mostly inaccurate information, 3—Accurate but with multiple errors, 4—Mostly accurate with minor errors, 5—Completely accurate; **Completeness**: 1—Totally incomplete, 2—Basic response provided but missing key information, 3—Neither incomplete nor complete, 4—Mostly complete answer providing most necessary information, 5—Totally complete and thorough answer; **Empathy:** 1—Very low empathy, 2—Low empathy, 3—Average empathy, 4—High empathy, 5—Very high empathy; **Understandability:** 1—Very high levels of medical jargon and flowery language, 2—High levels of medical jargon and flowery language, 3—Average levels of medical jargon and flowery language, 4—Low levels of medical jargon and flowery language, 5—Very low levels of medical jargon and flowery language; **Willingness to Send to Patient:** 1—No, I would definitely not send this response to a patient, 2—I would only consider sending this response to a patient after significant revisions, 3—I would send this response to a patient after several key modifications, 4—I would send this response to a patient after very minor modifications, 5—I would send this response to a patient without any changes or modifications.

**Fig. 3:**
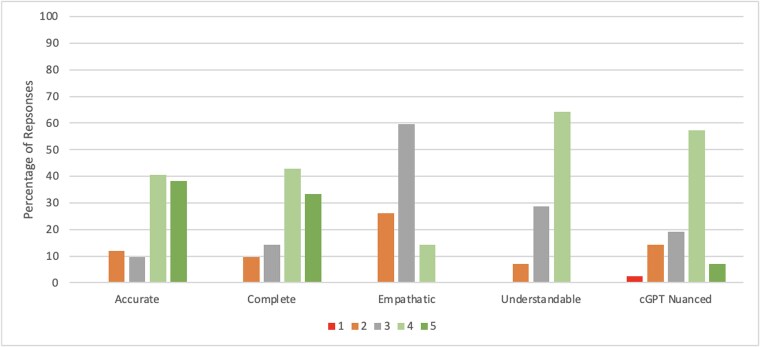
Expert rating of ChatGPT responses to clinical vignettes related to Barrett’s esophagus. † Likert scale ratings: **Accuracy:** 1—Completely inaccurate, 2—Mostly inaccurate information, 3—Accurate but with multiple errors, 4—Mostly accurate with minor errors, 5—Completely accurate; **Completeness**: 1—Totally incomplete, 2—Basic response provided but missing key information, 3—Neither incomplete nor complete, 4—Mostly complete answer providing most necessary information, 5—Totally complete and thorough answer; **Empathy:** 1—Very low empathy, 2—Low empathy, 3—Average empathy, 4—High empathy, 5—Very high empathy; **Understandability:** 1—Very high levels of medical jargon and flowery language, 2—High levels of medical jargon and flowery language, 3—Average levels of medical jargon and flowery language, 4—Low levels of medical jargon and flowery language, 5—Very low levels of medical jargon and flowery language; **Nuance/Specificity:** 1—The response is extremely general and does not address the specific clinical scenario at all, 2—The response is very general and is only slightly relevant to aspects of the clinical scenario, 3—The response is neither general nor specific to the provided clinical scenario, 4—The response is both nuanced and specific to most aspects of the provided clinical scenario, 5—The response is highly nuanced and addresses all aspects of the provided clinical scenario.

Gastroenterologists with expertise in BE rated the 29 ChatGPT-generated responses with an average accuracy rating of 4.01 (on a 5-point Likert Scale), with 79.3% of responses marked as either completely accurate or mostly accurate with minor errors (Figs 2 and 3). The average completeness rating of all responses was 4.08 out of 5, with 85.1% of all ChatGPT responses rated as either mostly or totally complete. The average empathy scores of all responses were rated lower at 2.97 out of 5, with only 20.7% of responses rated by gastroenterologists as containing high or very high levels of empathy. The average understandability rating of all responses was 3.62 out of 5, with 65.5% of responses rated as containing low or very low levels of medical jargon or flowery language. Tables 1 and 2 provide average ratings by the panel of gastroenterologists for each question, separated by question type. There were zero responses rated as completely inaccurate, totally incomplete, very low empathy, or with very high levels of medical jargon and flowery language (i.e. the lowest possible rating on each Likert scale). The 15 patient-centered responses received an average rating of 3.71 out of 5 by the gastroenterologists when asked if they were comfortable in sending ChatGPT responses to patients, with 64.4% of ChatGPT responses evaluated as acceptable to send with either very minor or no changes or modifications. Furthermore, 64.3% of responses to clinical vignettes were identified as nuanced and specific to either most or all of the clinical scenarios provided, with an average rating of 3.52 out of 5. Inter-rater reliability using Kendall’s coefficient of Concordance was 0.55, indicating a moderate to strong level of agreement among experts.

**Table 1:** Average ratings from three gastroenterologists of ChatGPT responses to patient-centered questions regarding Barrett’s esophagus

**Patient-centered questions**	**Accuracy**	**Completeness**	**Empathy**	**Excessive medical jargon**	**Comfortability in sending** **response to a patient**
**Q1.** What is Barrett’s esophagus?	4.00	4.67	3.33	3.00	3.67
**Q2.** What causes Barrett’s esophagus?	4.67	4.67	3.00	3.67	4.33
**Q3**. What are the risk factors of getting Barrett’s esophagus?	4.67	4.67	3.00	3.67	4.67
**Q4.** What are the symptoms of Barrett’s esophagus?	4.00	4.33	3.67	4.00	4.00
**Q5.** How Is Barrett’s esophagus diagnosed?	3.33	4.00	3.00	3.00	3.00
**Q6.** What is Barrett’s esophagus with dysplasia? Does it become cancer?	3.67	4.33	2.67	4.00	4.00
**Q7.** What lifestyle measures can I take to prevent Barrett’s esophagus from progressing?	3.67	4.00	3.00	4.00	3.00
**Q8.** Is there a diet that I should follow for Barrett’s esophagus?	4.33	4.33	3.00	4.33	4.33
**Q9.** Is there a medication that I can take to help my Barrett’s esophagus?	3.67	4.00	3.00	3.33	3.67
**Q10.** Can Barrett’s esophagus heal or reverse itself?	4.00	4.00	3.33	3.67	3.67
**Q11.** What are the treatment options for Barrett’s esophagus?	3.67	4.00	3.00	3.67	3.00
**Q12.** How often will I need endoscopy for Barrett’s esophagus?	3.67	4.00	3.33	3.00	3.67
**Q13.** What is endoscopic ablation and what should I expect?	3.67	3.33	2.67	4.33	3.00
**Q14.** My endoscopy pathology report states that I have low-grade dysplasia. What does that mean?	4.33	4.00	2.67	4.00	3.67
**Q15.** My endoscopy pathology report states that I have indefinite dysplasia. What does that mean?	4.33	4.00	3.00	3.33	4.00
**Average**	3.98	4.16	3.04	3.67	3.71

Likert scale ratings: **Accuracy:** 1—Completely inaccurate, 2—Mostly inaccurate information, 3—Accurate but with multiple errors, 4—Mostly accurate with minor errors, 5—Completely accurate; **Completeness**: 1—Totally incomplete, 2—Basic response provided but missing key information, 3—Neither incomplete nor complete, 4—Mostly complete answer providing most necessary information, 5—Totally complete and thorough answer; **Empathy:** 1—Very low empathy, 2—Low empathy, 3—Average empathy, 4—High empathy, 5—Very high empathy; **Excessive Medical Jargon:** 1—Very high levels of medical jargon and flowery language, 2—High levels of medical jargon and flowery language, 3—Average levels of medical jargon and flowery language, 4—Low levels of medical jargon and flowery language, 5—Very low levels of medical jargon and flowery language; **Comfortability:** 1—No, I would definitely not send this response to a patient, 2—I would only consider sending this response to a patient after significant revisions, 3—I would send this response to a patient after several key modifications, 4—I would send this response to a patient after very minor modifications, 5—I would send this response to a patient without any changes or modifications

**Table 2:** Average ratings from three gastroenterologists of ChatGPT responses to clinical vignette-style questions regarding Barrett’s esophagus

**Clinical vignette-style questions**	**Accuracy**	**Completeness**	**Empathy**	**Excessive medical jargon**	**Nuance/Specificity to clinical scenario**
**Q16.** Screening criteria for Barrett’s esophagus	5.00	5.00	3.67	4.00	4.33
**Q17.** Endoscopic diagnosis—abnormal mucosa <1 cm	3.33	3.33	2.67	3.00	2.33
**Q18.** Prague classification	4.33	3.67	3.00	3.67	3.67
**Q19.** Seattle protocol	4.00	3.33	2.67	3.00	3.33
**Q20.** Histological diagnosis of Barrett’s	4.33	4.33	2.67	3.00	4.00
**Q21.** Management of nondysplastic Barrett’s	4.00	4.67	3.00	3.33	3.67
**Q22.** Management of low-grade dysplasia	4.33	4.00	3.00	4.00	3.67
**Q23.** Management of indefinite dysplasia	4.33	4.00	2.67	4.00	3.33
**Q24.** Management of high-grade dysplasia	4.00	3.67	2.33	3.33	3.33
**Q25.** Management of Barrett’s with esophageal nodule	2.33	2.67	2.33	3.33	2.33
**Q26.** Need for repeat screening after a negative endoscopy	5.00	5.00	3.67	4.00	4.00
**Q27.** Anti-reflux surgery and risk of developing esophageal cancer	4.67	5.00	3.00	3.67	4.33
**Q28.** Continued surveillance after CEIM for baseline diagnosis of low-grade dysplasia	3.33	3.33	3.00	4.00	3.67
**Q29.** Continued surveillance after CEIM for baseline diagnosis of high-grade dysplasia	3.67	4.00	2.67	3.67	3.33
**Average**	4.05	4.00	2.88	3.57	3.52

CEIM, complete eradication of intestinal metaplasia. Likert-scale ratings: **Accuracy:** 1—Completely inaccurate, 2- Mostly inaccurate information, 3- Accurate but with multiple errors, 4- Mostly accurate with minor errors, 5- Completely accurate; **Completeness**: 1- Totally incomplete, 2—Basic response provided but missing key information, 3—Neither incomplete nor complete, 4- Mostly complete answer providing most necessary information, 5- Totally complete and thorough answer; **Empathy:** 1—Very low empathy, 2—Low empathy, 3—Average empathy, 4—High empathy, 5—Very high empathy; **Excessive Medical Jargon:** 1—Very high levels of medical jargon and flowery language, 2—High levels of medical jargon and flowery language, 3—Average levels of medical jargon and flowery language, 4—Low levels of medical jargon and flowery language, 5 -Very low levels of medical jargon and flowery language; **Nuance/Specificity:** 1—The response is extremely general and does not address the specific clinical scenario at all, 2—The response is very general and is only slightly relevant to aspects of the clinical scenario, 3—The response is neither general nor specific to the provided clinical scenario, 4—The response is both nuanced and specific to most aspects of the provided clinical scenario, 5—The response is highly nuanced and addresses all aspects of the provided clinical scenario.

**Fig. 4:**
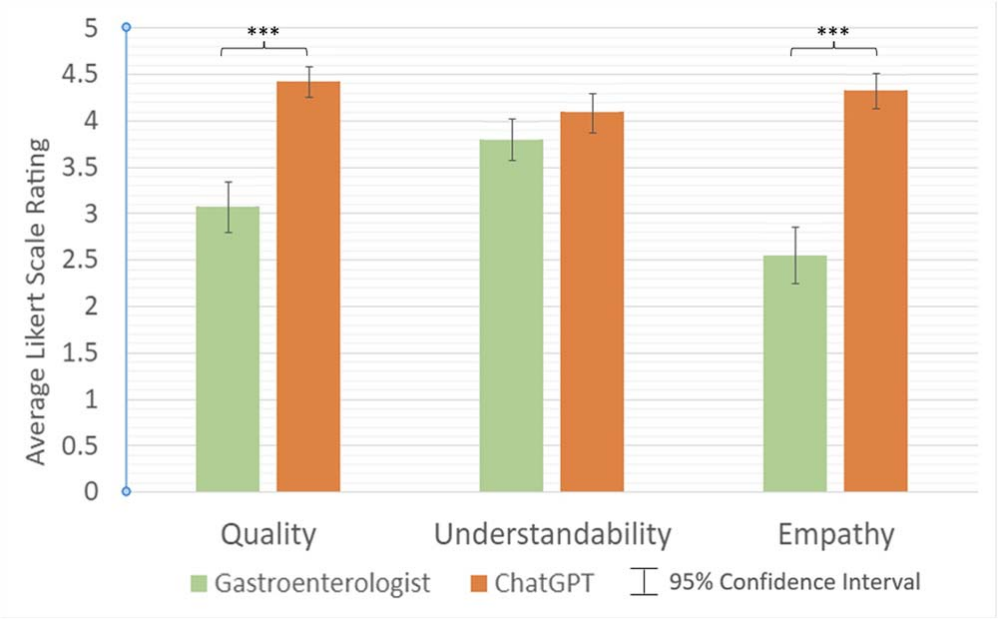
Patient average ratings of gastroenterologist versus ChatGPT responses. *** indicates statistical significance (*p* < 0.05). † Likert scale ratings: **Quality:** 1—Very low quality, 2—Low quality, 3—Average quality, 4—High quality, 5—Very high quality; **Understandability:** 1—Very difficult to understand, 2—Difficult to understand, 3—Neither easy nor difficult to understand, 4—Easy to understand, 5—Very easy to understand; **Empathy:** 1—Very low empathy, 2—Low empathy, 3—Average empathy, 4—High empathy, 5—Very high empathy.

In the second part of the study, the patient panel found ChatGPT responses to be of significantly higher quality compared to physician responses (4.42 vs. 3.07 out of 5, *p* < 0.001), with only 35.5% of ratings for gastroenterologist responses indicating high or very high quality, compared to 97.8% for ChatGPT responses (Fig. 4). Patients also found ChatGPT responses to have significantly higher empathy (4.33 vs. 2.55 out of 5, *p* < 0.001) when compared to gastroenterologist-generated responses. In total, 95.6% of the ratings for ChatGPT responses demonstrated high or very high empathy levels, compared to only 13.3% for gastroenterologist responses.

When assessing understandability, patients found both ChatGPT and gastroenterologist responses to be coherent, with average scores of 4.09 and 3.8 out of 5, respectively (*p* = 0.085). 77.8% of the ratings for ChatGPT responses were evaluated as easy or very easy to understand, compared to 66.6% for ratings of physician responses. Table 3 summarizes the average patient rating of both human and AI responses to each question. When asked which of the two responses (AI vs. gastroenterologist) they preferred, patients selected ChatGPT responses 84.4% of the time. Regarding readability, ChatGPT responses showed an average Flesch–Kincaid reading level of 14 with an average reading ease score of 25.76, indicating a college-graduate reading level. Similarly, the average Flesch–Kincaid reading level of all gastroenterologist responses was 14 with an average reading ease score of 30.

**Table 3:** Average ratings from patients of ChatGPT versus gastroenterologist responses regarding patient-centered Barrett’s esophagus questions

**Patient-centered questions**	**Quality/Satisfaction**		**Understandability**		**Empathy**	
	GI	ChatGPT	GI	ChatGPT	GI	ChatGPT
**Q1.** What is Barrett’s esophagus?	3.67	4.67	3.67	4.67	2.67	4.67
**Q2.** What causes Barrett’s esophagus?	3.33	4.67	4.33	4.33	2.67	4.00
**Q3.** What are the risk factors of getting Barrett’s esophagus?	3.67	4.67	4.00	4.33	2.67	4.33
**Q4.** What are the symptoms of Barrett’s esophagus?	3.33	5.00	4.00	4.33	2.00	4.33
**Q5.** How Is Barrett’s esophagus diagnosed?	3.33	4.67	3.33	4.33	2.67	4.67
**Q6.** What is Barrett’s esophagus with dysplasia? Does it become cancer?	3.67	4.67	4.33	4.00	3.00	4.33
**Q7.** What lifestyle measures can I take to prevent Barrett’s esophagus from progressing?	3.33	4.67	3.67	4.33	2.33	4.67
**Q8.** Is there a diet that I should follow for Barrett’s esophagus?	1.33	4.33	4.33	4.33	1.67	4.33
**Q9.** Is there a medication that I can take to help my Barrett’s esophagus?	1.67	4.33	4.33	4.00	2.00	4.33
**Q10.** Can Barrett’s esophagus heal or reverse itself?	2.67	4.33	3.67	4.00	2.00	4.33
**Q11.** What are the treatment options for Barrett’s esophagus?	3.00	4.33	3.67	4.00	3.33	4.33
**Q12.** How often will I need endoscopy for Barrett’s esophagus?	3.67	4.33	3.67	3.67	3.33	4.33
**Q13.** What is endoscopic ablation and what should i expect?	3.33	3.67	3.67	3.33	2.67	3.67
**Q14.** My endoscopy pathology report states that I have low-grade dysplasia. What does that mean?	2.67	4.00	3.33	3.67	2.67	4.33
**Q15.** My endoscopy pathology report states that I have indefinite dysplasia. What does that mean?	3.33	4.00	3.00	4.00	2.67	4.33
**Average**	3.07	4.42	3.80	4.09	2.56	4.33
***P*-value**	<0.001^*^		0.085		<0.001^*^	

^*^indicates statistical significance, *p* < 0.05.

## DISCUSSION

As large language models (LLMs) continue to be refined and improved, there has been great interest in their application and integration into the medical field.^20–22^ Several studies have looked at the use of various versions of ChatGPT within the field of gastroenterology with variability and inconsistency in its performance. ChatGPT has been shown to accurately answer patient-centered questions in topics such as colonoscopies, cirrhosis, hepatocellular carcinoma, pancreatic cancer, and GERD, while other topics such as eosinophilic esophagitis, inflammatory bowel disease, and colorectal cancer screening highlight some of ChatGPT’s limitations.^9–16^^,^^23^ For instance, Pereyra *et al*. reported in a study that ChatGPT performance was both inaccurate and inconsistent when determining appropriate colorectal cancer screening recommendations on realistic clinical vignettes.^16^

Regarding BE, we found that ChatGPT provides adequate medical knowledge when given clinical vignettes as reviewed by three expert gastroenterologists. There were no glaring inaccuracies in ChatGPT responses; however, the content errors that ChatGPT did display involved providing non-specific recommendations that, while not harmful, were not clearly applicable, such as recommending yoga and deep breathing for stress management as lifestyle measures to prevent BE progression. ChatGPT also provided some outdated medical recommendations including esophagectomy for high-grade dysplasia and failed to mention newer therapeutic interventions like radiofrequency ablation (RFA) or endoscopic submucosal dissection. Novel therapies may handicap ChatGPT’s capability in providing up-to-date recommendations for patients as the wealth of literature is not as abundant as established treatments. Overall, ChatGPT’s ability to provide accurate medical information likely is dependent on the subject matter and this may partly explain conflicting results in the literature.^14–16^^,^^20^^,^^21^ Given that ChatGPT synthesizes information that is readily available on the internet, it may be able to provide higher-quality responses on medical topics with clear-cut society guidelines and a wealth of published literature. Conversely, ChatGPT may have difficulty with medical topics that involve rapidly evolving recommendations or those requiring high levels of clinical experience or nuance, which are limitations previously observed.^22^ Additionally, although the physician panel in our study found the ChatGPT responses to contain high levels of accuracy, one systematic literature review by Shung *et al*. found generally low accuracy levels in responses to gastroenterology and hepatology-related questions by LLMs, including ChatGPT 4.0.^23^

Our use of frequently asked patient questions simulates an interaction between patients and ChatGPT, which overall showed adequate medical knowledge and, interestingly, was preferred by patients when compared to gastroenterologist-generated responses. Compared to gastroenterologists, ChatGPT provided longer and more comprehensive responses, which patients favored in quality, empathy, and overall preference. This is congruent with a previous study by Ayers *et al.*, demonstrating that in a forum of patient question queries, physicians overall rated chatbot responses as having higher quality and empathy.^12^ Given a busy clinician’s time constraints, providing long and extensive responses may not be feasible for every patient inquiry. As EHR^16^ inboxes have been identified as a contributor to ever-increasing physician burnout, we highlight that ChatGPT has the potential to improve physician workflow and efficiency.^19^

A recently published study by Garcia *et al*. demonstrated that when providers utilized draft replies to patient portal messages generated by an EHR-integrated LLM, there was no significant change in reply action time, write time, or read time.^24^ Although results from their study and ours cannot be directly compared given differences in methodology, outcomes measured, and combination of different specialties (predominantly family medicine and general internal medicine vs. a specific topic in gastroenterology), it is interesting to note that in their subgroup analysis, gastroenterology, and hepatology nurses had higher draft utilization and a trend toward time saved and positive net promoter scores.^24^ As our study found that >80% of ChatGPT responses were appropriate to be sent with minimal change and that there was a clear preference by patients for ChatGPT responses over gastroenterologist-generated responses; there is a promising potential value of using AI generative tools in specific practice patterns and workflows within gastroenterology. For example, many clinicians today often use premade responses or have their support staff draft replies. Use of an AI-assisted approach could potentially help relieve work burden and give back time to physicians and clinical staff to focus on other tasks. Having clinical staff review and modify AI-written drafts would allow for more consistent responses and improve communication with patients.

The results and themes from previous studies and ours suggest that in its current iteration, ChatGPT cannot perform autonomously and requires healthcare provider monitoring and input. However, a healthcare provider harnessing the power of AI ‘provider–AI hybrid’ could potentially be the answer for better care while reducing burnout.^25^ This has already been observed in previous studies, as utilization of AI-generated draft replies to patient portal messages resulted in improvement in assessments of burden and burnout among providers in gastroenterology and hepatology.^24^ As seen in prior studies, such as in ChatGPT and EOE, we found that ChatGPT provided responses at a college graduate reading level. Importantly, the American Medical Association recommends that medical information be below a sixth-grade reading level to best promote health literacy.^15^^,^^26^ However, our evaluation of gastroenterologist-generated responses also displayed a college graduate reading level, which highlights the need for both physicians and AI to tailor medical communication for each patient to ensure readability. A potential solution that was not pursued in this study is to specifically add additional prompting to the LLM so that responses are tailored to a certain reading level.

One potential limitation of any study using ChatGPT is the inherent bias of its training data. The model’s responses are based on a diverse but uncontrolled data set of information gathered from the internet, which is uncurated and may include false or outdated information. To address this challenge, there is ongoing discussion about the need to utilize refinement techniques, such as reinforcement learning from human feedback, to increase LLM accuracy.^27^ Other limitations to our study include its observational nature and relatively low sample size of patients. We also acknowledge that our study is limited to BE and our conclusions may not be applicable to other medical conditions. Additionally, although our study is novel in its comparison of empathy levels between ChatGPT and gastroenterologists, empathy is a nebulous concept and use of a Likert scale may not fully capture the complexity and nuance of effective patient communication. Lastly, ChatGPT boasts distinct responses to identical prompts, which limits standardization of information for patients. This is not a clear limitation as a patient aid, as different human providers may display similar incongruity when faced with identical prompts. Each question was submitted twice to ChatGPT, and we found that although the formatting of responses varied with each iteration, the content remained consistent with each query. It is worth noting that further updates in ChatGPT programming are ongoing and the responses generated in this particular version may not reflect results derived from subsequent versions of ChatGPT.

Overall, our investigation comprehensively elucidates a role for ChatGPT regarding patient inquiries on Barrett’s esophagus. We found that ChatGPT provides adequate medical knowledge when evaluated by expert gastroenterologists and overall, its responses were preferred by patients when compared to gastroenterologist-generated responses. To our knowledge, this is the first comprehensive study within a specific topic in gastroenterology demonstrating that ChatGPT is medically accurate and preferred by patients when compared to human gastroenterologists. Given inconsistencies on prior ChatGPT performance on various topics in GI, it is important that more of these studies are performed on different topics until the technology has advanced enough to consistently provide accurate and up to date medical information. Further studies are needed to reassess medical accuracy, patient satisfaction and characterize time and energy spent on EHR inbox when ChatGPT is implemented as a physician aid.

## Study highlights

WHAT IS KNOWN:

- Barrett’s esophagus is one of the most common gastrointestinal disorders that has a high burden on the US healthcare system.
- ChatGPT has been shown to be a proven tool for patient education in common gastrointestinal disorders such as colon cancer and cirrhosis.

WHAT IS NEW HERE:

- ChatGPT 4.0 can provide accurate and comprehensive information about Barrett’s esophagus.
- Patients prefer ChatGPT responses over gastroenterologists in terms of quality and empathy.
- ChatGPT 4.0 can be potentially used as an adjunctive tool for physicians to provide real-time, high-quality information about Barrett’s esophagus to patients.

## Notes

**Specific author contributions**: All authors have reviewed and approved this final draft of the manuscript. Frances Dang (Conceptualization, Data curation, Investigation, Methodology, Project administration, Supervision, Visualization, Writing—original draft, Writing—review & editing), Joshua Kwon (Data curation, Investigation, Methodology, Writing—original draft, Writing—review & editing), Andy Lin (Data curation, Formal analysis, Investigation, Methodology, Writing—original draft, Writing—review & editing), Shoujit Banerjee (Data curation, Investigation, Methodology, Writing—original draft, Writing—review & editing), Trevor McCracken (Data curation, Investigation, Writing—original draft, Writing—review & editing), Amirali Tavangar (Formal analysis, Project administration, Writing—review & editing), Shravani R. Reddy (Data curation, Investigation, Writing—review & editing), Alyssa Y. Choi (Investigation, Writing—review & editing), Jennifer Phan (Investigation, Writing—review & editing), Jeffrey D. Mosko (Investigation, Writing—review & editing), Samir C. Grover (Investigation, Writing—review & editing), Tyler Berzin (Investigation, Writing—review & editing), and Jason Samarasena (Conceptualization, Data curation, Investigation, Methodology, Supervision, Visualization, Writing—review & editing)

**Conflicts of interests**: The authors certify that we have no financial arrangements (e.g. consultancies, stock ownership, equity interests, patent-licensing arrangements, research support, honoraria) with a company whose product figures prominently in this manuscript or with a company making a competing product.

## Appendix I: List of patient-centered questions and clinical vignettes on Barrett’s Esophagus

*Common frequently asked questions posed by a patient newly diagnosed with Barrett’s Esophagus:*

1. What is Barrett’s esophagus?
2. What causes Barrett’s esophagus?
3. What are the risk factors of getting Barrett’s esophagus?
4. What are the symptoms of Barrett’s esophagus?
5. How is Barrett’s esophagus diagnosed?
6. What is Barrett’s esophagus with dysplasia? Does it become cancer?
7. What lifestyle measures can I take to prevent Barrett’s esophagus from progressing?
8. Is there a diet that I should follow for Barrett’s esophagus?
9. Is there a medication that I can take to help my Barrett’s esophagus?
10. Can Barrett’s esophagus heal or reverse itself?
11. What are the treatment options for Barrett’s esophagus?
12. How often will I need endoscopy for Barrett’s esophagus?
13. What is endoscopic ablation, and what should I expect?
14. My endoscopy pathology report states that I have low-grade dysplasia. What does that mean?
15. My endoscopy pathology report states I have indefinite dysplasia. What does that mean?

*Clinical vignettes and relevant topic being reviewed:*

1. **Screening criteria for Barrett’s** 
  - Q: A 52-year-old Caucasian man is evaluated for GERD in your office. He has a longstanding history of GERD for over 20 years. He also uses cigarettes actively, smoking 1 pack per day for the last 28 years. Should this patient undergo endoscopic screening for Barrett’s esophagus? What are the criteria for indications for Barrett’s screening?
2. **Endoscopic diagnosis—abnormal mucosa <1cm** 
  - Q: A 55-year old male patient with chronic GERD undergoes upper endoscopy for screening of Barrett’s esophagus. During the endoscopy, an irregular z-line is seen at the gastroesophageal junction, but there is no abnormal mucosa extending 1cm above the z-line. Should biopsies be taken?
3. **Prague classification** 
  - Q: A patient undergoes upper endoscopy to evaluate chronic heartburn. The endoscopy report states there is presence of C3M4 Barrett’s esophagus. What does this mean?
4. **Seattle protocol** 
  - Q: If Barrett’s esophagus is suspected on endoscopy, how should biopsies be done?
5. **Histological diagnosis of Barrett’s** 
  - Q: What are the classic biopsy findings of the esophagus that may be used to identify Barrett esophagus microscopically?
6. **Management of nondysplastic Barrett’s** 
  - Q: A 60-year-old Caucasian man is here for a follow-up visit for management of his chronic reflux. The patient undergoes endoscopy, which shows a 2 cm section of Barrett esophagus but without dysplasia on biopsy. He has been taking a proton pump inhibitor daily, and his symptoms are well controlled on this medication. When should this patient undergo surveillance endoscopy?
7. **Management of low-grade dysplasia** 
  - Q: A 60-year-old Caucasian man is here for a follow-up visit for management of his chronic reflux. The patient undergoes endoscopy which shows a 2 cm section of Barrett esophagus with low-grade dysplasia. He has been taking a proton pump inhibitor daily, and his symptoms are well controlled on this medication. What should be recommended to this patient?
8. **Management of indefinite dysplasia** 
  - Q: A 60-year-old Caucasian man is here for a follow-up visit for management of his chronic reflux. The patient undergoes endoscopy, which shows a 2 cm section of Barrett’s esophagus that was indefinite for dysplasia. He is not taking any medications at this time and complains of frequent heartburn. What should be recommended to this patient?
9. **Management of high-grade dysplasia** 
  - Q: A 60-year-old Caucasian man is here for a follow-up visit for management of his chronic reflux. The patient undergoes endoscopy, which shows a 2 cm section of Barrett’s esophagus with high-grade dysplasia. He has been taking a proton pump inhibitor daily, and his symptoms are well controlled on this medication. What should be recommended to this patient?
10. **Management of Barrett’s with esophageal nodule** 
  - Q: A 52-year-old Caucasian man undergoes upper endoscopy for evaluation of chronic reflux. At the gastroesophageal junction, an irregular b z-line is seen with circumferential salmon colored mucosa extending 4 cm above the z-line, concerning for Barrett’s esophagus. A large nodule is seen in the esophagus within the suspected Barrett’s esophagus. What is the next best step in management?
11. **Need for repeat screening after a negative endoscopy** 
  - Q: A 55-year-old male patient with chronic GERD that is well controlled with lifestyle modifications undergoes screening endoscopy to evaluate for Barrett’s esophagus. The endoscopy is unremarkable with a normal esophagus. Does this patient need further screening for upper endoscopies?
12. **Anti-reflux surgery and risk of developing esophageal cancer** 
  - Q: A 55-year-old male with chronic reflux and recently diagnosed nondysplastic Barrett’s esophagus is seen in the clinic. He has well-controlled GERD symptoms on a daily PPI. Should this patient pursue anti-reflux surgery to reduce his risk for developing esophageal cancer?
13. **Recommendations of continued surveillance after CEIM for baseline diagnosis of LGD** 
  - Q: A 55-year-old male patient is found to have a 3 cm segment of Barrett’s esophagus with low-grade dysplasia on a screening endoscopy. He returns for a repeat endoscopy, and radiofrequency ablation is performed successfully on the Barrett’s mucosa. A follow-up endoscopy shows evidence of complete eradication of intestinal metaplasia (CEIM). What should be this patient’s surveillance endoscopy schedule?
14. **Recommendations of continued surveillance after CEIM for baseline diagnosis of HGD** 
  - Q: A 55-year-old male patient is found to have a 3 cm segment of Barrett’s esophagus with high-grade dysplasia on a screening endoscopy. He returns for a repeat endoscopy and radiofrequency ablation is performed successfully on the Barrett’s mucosa. A follow up endoscopy shows evidence of complete eradication of intestinal metaplasia (CEIM). What should be this patient’s surveillance endoscopy schedule?

## Appendix II: Gastroenterologist Grading Scale of ChatGPT Responses

The following scales will be applicable to all 30 responses generated by ChatGPT:

(A) Please rate how accurate you found this response to be:

- Completely inaccurate
- Mostly inaccurate information
- Accurate but with multiple errors
- Mostly accurate with minor errors
- Completely accurate

(B) Please rate how thorough and complete you found this response to be:

- Totally incomplete
- Basic response provided but missing key information
- Neither incomplete nor complete
- Mostly complete answer providing most necessary information
- Totally complete and thorough answer

(C) Please rate how empathetic and personable you found this answer to be:

- Very low empathy
- Low empathy
- Average empathy
- High empathy
- Very high empathy

(D) Please rate the level of flowery language or excessive medical jargon in each response:

- Very high levels of medical jargon and flowery language
- High levels of medical jargon and flowery language
- Average levels of medical jargon and flowery language
- Low levels of medical jargon and flowery language
- Very low levels of medical jargon and flowery language

*The following question was only asked for patient-centered responses (Questions 1–15):*

(E) Would you feel comfortable sending this response to a patient?

- No, I would definitely not send this response to a patient
- I would only consider sending this response to a patient after significant revisions
- I would send this response to a patient after several key modifications
- I would send this response to a patient after very minor modifications
- I would send this response to a patient without any changes or modifications

*The following question was only asked for clinical vignette responses (Questions 16–29):*

(F) In your opinion, please indicate if the response provided by ChatGPT is nuanced and specific to the provided clinical scenario:

- The response is extremely general and does not address the specific clinical scenario at all
- The response is very general and is only slightly relevant to aspects of the clinical scenario
- The response is neither general nor specific to the provided clinical scenario
- The response is both nuanced and specific to most aspects of the provided clinical scenario
- The response is highly nuanced and addresses all aspects of the provided clinical scenario

## Appendix III: Patient grading scale of ChatGPT versus gastroenterologist responses to questions regarding Barrett’s esophagus

*These following questions were asked for both a “Response A” and “Response B,” which were randomized between ChatGPT and a gastroenterologist’s response to a given question.*

1. Please rate the overall quality:
  - Very low quality
  - Low quality
  - Average quality
  - High quality
  - Very high quality
2. Please rate how easy to understand you found the response:
  - Very difficult to understand
  - Difficult to understand
  - Neither easy nor difficult to understand
  - Easy to understand
  - Very easy to understand
3. Please rate how empathetic and personable you found the response:
  - Very low empathy
  - Low empathy
  - Average empathy
  - High empathy
  - Very high empathy

*Additionally, the questions below were also asked regarding each set of responses.*

- Which response did you prefer?
- Response A
- Response B
- Please identify if you believe if the answer was produced by ChatGPT (artificial intelligence) or by a human gastroenterology physician.
- ChatGPT
- Human Gastroenterologist

## Appendix IV: List of US Hospital Centers and Barrett’s Esophagus Patient Support and Awareness Groups

1. FAQ: Barrett’s esophagus. Massachusetts General Hospital. Accessed September 7, 2024.

 https://www.massgeneral.org/digestive/treatments-and-services/barretts/faq.

2. Barrett’s esophagus. Cleveland Clinic. Accessed September 7, 2024. https://my.clevelandclinic.org/health/diseases/14432-barretts-esophagus

3. Barrett’s Esophagus Awareness. Facebook. Accessed September 7, 2024. https://www.facebook.com/groups/barrettesophagusawareness.

4. Barrett’s esophagus. AGA GI Patient Center. Accessed September 7, 2024. https://patient.gastro.org/barretts-esophagus/

5. Enzinger PC. Five things you need to know about Barrett’s esophagus. Dana-Farber Cancer Institute. November 22, 2019. Accessed September 7, 2024. https://blog.dana-farber.org/insight/2019/11/five-things-you-need-to-know-about-barretts-esophagus/

6. Stallard J. What should I know about Barrett’s esophagus and risk for esophageal cancer? Memorial Sloan Kettering Cancer Center. July 10, 2020. Accessed September 7, 2024. https://www.mskcc.org/news/what-should-know-about-barrett-s-esophagus-and-risk-esophageal

7. Azodo IA, Romero Y. Barrett’s Esophagus. American College of Gastroenterology. January 2006. Accessed September 7, 2024. https://gi.org/topics/barretts-esophagus/
